# Average performance and performance variability on a brief functional fine motor test reflect distinct behavioral constructs in aging

**DOI:** 10.3389/fnagi.2026.1957422

**Published:** 2026-09-22

**Authors:** Chung-Fu Sun, Kylie B. Tomlin, Jennapher Lingo VanGilder, Andrew Hooyman, Alexandra M. Reed, Jessica L. Trevino, Sydney Y. Schaefer

**Affiliations:** 1 School of Biological and Health Systems Engineering, Arizona State University, Tempe, AZ, United States; 2 Department of Imaging Sciences, St. Jude Children's Research Hospital, Memphis, TN, United States; 3 Department of Physical Therapy, Chapman University, Irvine, CA, United States; 4 Arizona Alzheimer’s Consortium, Phoenix, AZ, United States

**Keywords:** functional fine motor, grip strength, grooved pegboard, intrasubject variability, memory test

## Abstract

Prior work in older adults has developed a brief functional fine motor test, which involves repeated timed trials of an upper-limb task. Performance has been associated with global and domain-specific neuropsychological tests as well as putative markers of Alzheimer’s disease when scored using variability in trial time; however, the extent to which task performance depends on motor ability has not been established nor quantified. This retrospective study examined whether mean trial time and trial time variability (measured as intrasubject standard deviation in trial time) were differentially associated with standard assessments of motor and cognitive function. Forty-eight older adults with no functional impairment completed the functional fine motor test, which was compared to grooved pegboard test performance, grip strength, and Rey–Osterrieth complex figure test (RCFT) delayed recall. Generalized linear models included age, sex, education, and both scoring methods on the functional fine motor test (mean trial time and trial time variability) as predictors. Longer mean trial time was associated with slower grooved pegboard performance and lower grip strength, whereas trial time variability was not associated with either standard motor assessment. These findings, along with patterns of partial correlations, suggest that mean trial time may predominantly reflect motor function while trial time variability does not. Together, these findings highlight the value of considering both mean performance and performance variability in tasks that include multiple trials, offering an efficient approach to assessing multiple domains in older adults.

## 1 Introduction

Alzheimer’s disease (AD) and related dementias are associated with changes in both cognitive and motor functioning (Buchman and Bennett, 2011; Oveisgharan et al., 2024). Although cognitive symptoms are often the primary clinical concern, motor features such as slower movement, reduced fine motor dexterity, impaired coordination, and lower strength are also commonly observed with normal aging and neurodegenerative disease progression (Koppelmans et al., 2024). However, existing performance-based assessments of cognition (e.g., clock drawing) and daily functioning (e.g., UCSD Performance-Based Skills Assessment, Independent Living Scales) inherently involve motor behavior, but most do not necessarily consider nor dissociate the extent of motor impairment in the overall score. Of note, the Trail Making Tests (TMT) A and B can be compared to dissociate processing speed from executive function, whereby clinicians may subtract the completion time for TMT-A from that of TMT-B (Strauss et al., 2006; Kopp et al., 2015), but this approach requires the administrating and completion of two separate tests (versions A and B) and is typically done for research purposes.

Although some motor assessments involve cognitive demands in addition to motor execution (Buchman and Bennett, 2011; Solaro et al., 2024), they are not necessarily designed to evaluate traditional cognitive domains (e.g., memory, visuospatial ability) nor the integration of cognition into real-world daily activities (i.e., functional cognition) (Giles et al., 2025). However, recent clinical practice guidelines recommend that primary care clinicians conduct a structured multi-domain evaluation spanning cognitive, neuropsychiatric, sensorimotor, and functional (i.e., ADL) domains in older adults (Atri et al., 2025). Given the time constraints and competing demands experienced by clinicians (Porter et al., 2023), there is a critical need for brief, efficient screening approaches that maximize clinically relevant information across these domains during routine encounters, without redundancy (Mattke et al., 2023). To this end, a functional fine motor test has been developed, in which multiple trials of a timed functional task are performed in a row (scooping raw beans with a spoon and transporting them to various targets in a sequence) (Schaefer and Duff, 2015). Critically, this test has been validated against standard neuropsychological assessments of verbal memory, visuospatial function, and processing speed when scored as the variability in trial time across consecutive trials (Hooyman et al., 2023). This measure of task performance has also been shown to be sensitive to atrophy in cortical areas associated with these cognitive domains rather than sensorimotor areas (Malek-Ahmadi et al., 2023), suggesting that variability in trial time reflects cognitive rather than motor abilities. This interpretation is consistent with prior studies showing that greater intraindividual variability on repeated cognitive and motor assessments is associated with cognitive impairment and early stages of neurodegenerative diseases in older adults (Costa et al., 2019; Koppelmans et al., 2023; Scott et al., 2023). However, the extent to which the functional fine motor test depends on motor abilities has not been evaluated.

This retrospective analysis leveraged existing available data to therefore examine whether another scoring method other than variability in trial time may show stronger associations with motor function (e.g., speed, dexterity). Based on prior work, we hypothesized that mean trial time would show stronger associations with standard measures of motor function (i.e., Grooved Pegboard and grip strength, representing manual dexterity and visuomotor coordination versus force production, respectively) (Trites, 1989; Cui et al., 2021), whereas trial time variability would show stronger associations with measures of cognitive function (specifically, visuospatial memory). The delayed recall portion of the Rey-Osterrieth Complex Figure Test (RCFT) was selected as the cognitive measure of interest because visuospatial memory has previously been associated with performance variability and motor learning on this functional fine motor task (Hooyman et al., 2023; Malek-Ahmadi et al., 2023). These measures were selected based on their availability in the prior dataset.

## 2 Methods

### 2.1 Participants

Data from 48 older adults from an existing study cohort were included in this retrospective analysis (mean ± SD age = 70.17 ± 6.49 years; 30 women; 16.10 ± 2.44 years of education). All participants reported no functional impairment (defined as “full independence” on the Katz Index of Independence in Activities of Daily Living (Katz et al., 1963)). All but one participant also had Wechsler Adult Intelligence Scale Version IV (WAIS-IV) Perceptual Reasoning Index (PRI) composite scores within the normative range (mean: 100; range: 85 to 115); sensitivity analyses without this participant (PRI composite score = 84) are included in the Supplementary material. Participant characteristics are summarized in Table 1. All participants provided informed consent prior to participation in the primary study in compliance with local institutional requirements; the primary study protocol was approved by the Arizona State University Institutional Review Board (STUDY00004214).

**Table 1:** Participant characteristics.

Characteristics	Cognitively unimpaired older adults *n* = 48
Age	70.17 ± 6.49
Education (years)	16.10 ± 2.44
Sex (# Male/Female)	18/30
Mean trial time (s)	54.32 ± 9.44
Trial time variability (s)	5.50 ± 2.28
Grooved pegboard time, nondominant hand (s)	107.00 ± 50.98
Grip strength, nondominant hand (kg)	26.64 ± 10.21
Rey-Osterrieth Complex Figure Test, Delayed Recall	15.74 ± 7.05

All values are mean ± SD unless otherwise indicated. Sex is presented as male/female. SD = standard deviation; RCFT = Rey-Osterrieth Complex Figure Test.

### 2.2 Assessments

#### 2.2.1 Functional fine motor test

Analyses included participants’ performance on six repeated timed trials of a functional upper extremity task (Malek-Ahmadi et al., 2023). To summarize, participants used a standard spoon with the nondominant hand to move two raw kidney beans at a time from a central home cup to three target cups in a repeated sequence, returning to the home cup after each transfer. An image of the test set-up is provided in Supplementary Figure S1. Each trial consisted of 15 movements, and trial time was recorded as the elapsed time required to complete the sequence. From this, two scoring methods were calculated for each participant: mean trial time and trial time variability. Mean trial time was calculated as the average trial time across the six trials, with higher values indicating slower average performance. This measure was selected over the trial time of a single trial (e.g., the first trial) because single-trial measures tend to yield unstable estimates of central performance and lower test–retest reliability than averaged measures, thereby limiting their clinical utility (Luque-Casado et al., 2021; Mokkink et al., 2023); however, Supplementary Tables S1–S3 includes analyses for the first trial for comparison. Trial time variability was calculated as the standard deviation in trial time across the six trials, with higher values indicating greater trial-to-trial variability (Malek-Ahmadi et al., 2023).

#### 2.2.2 Grooved pegboard

The Grooved Pegboard Test is used clinically to assess fine motor speed, manual dexterity, and visual-motor coordination (Trites, 1989). According to standardized test instructions, participants placed a total of 25 grooved pegs one at a time into slotted holes as quickly as possible. This test requires participants to manipulate and often rotate the peg with their fingers to match the orientation of the slot. Performance was scored as the completion time to insert all 25 pegs, with longer completion times indicating worse motor performance. Because the nondominant hand is used in the functional fine motor test, only Grooved Pegboard performance for the nondominant hand was collected.

#### 2.2.3 Grip strength

Participants were assessed using a Baseline® hydraulic hand dynamometer with grip strength recorded in kilograms (kg). Participants performed three trials with each hand, alternating between the dominant and nondominant hands. The mean value for each hand was calculated, with higher values indicating stronger (better) grip strength. For reasons stated above, only nondominant hand grip strength was analyzed here.

#### 2.2.4 Rey–Osterrieth complex figure test

The Rey–Osterrieth Complex Figure Test (RCFT) was used to assess visuospatial construction and visual memory (Rey, 1941; Osterrieth, 1944; Meyers and Meyers, 1995). Per the test instructions, participants first copied the copyrighted complex geometric figure in the test booklet, and then reproduced the figure from memory after 5 min (immediate recall) and again after 30 min (delayed recall). Based on prior work (Lingo VanGilder et al., 2022), the delayed recall score was used as the cognitive outcome in the present analyses, with higher scores indicating better visuospatial memory.

#### 2.2.5 Order of assessment

In the original study protocol, all participants completed the assessments in the same order. On the evaluation day (considered baseline in the prior study), participants completed the RCFT copy and immediate recall portions (not analyzed here), and were then administered the Grooved Pegboard Test, grip strength, and then the RCFT delayed recall portion. Participants then completed the functional fine motor test as described here 1 week later.

### 2.3 Statistical analyses

Statistical analyses were conducted in R (R 4.5.2; R Foundation for Statistical Computing, Vienna, Austria). All outcome distributions were examined using normality tests and visual inspection of distributional plots prior to model selection. The primary dependent variables for motor function were Grooved Pegboard completion time and nondominant hand grip strength, both of which had right-skewed distributions and were therefore analyzed using Gamma generalized linear models with log links. The primary dependent variable for cognition (memory) was the RCFT delayed recall, which was analyzed using a Gaussian generalized linear model with an identity link because the outcome data were approximately normally distributed. All models included age, sex, education, mean trial time, and trial time variability as predictors. To visualize the observed relationships, scatterplots of the motor and cognitive assessments against mean trial time and trial time variability were generated (Supplementary Figure S2). Mean trial time and trial time variability were entered into the same models to determine whether each scoring method was associated with motor or cognitive outcomes after accounting for the other scoring method. Statistical significance was evaluated using an alpha level of 0.05. A sensitivity power analysis was conducted using G*Power version 3.1.9.6 (Faul et al., 2009) for a fixed-effects multiple regression model testing the incremental contribution of one predictor. To further summarize the covariate-adjusted association pattern among study variables, Pearson partial correlations were computed for age, sex, education, mean trial time, trial time variability, Grooved Pegboard completion time, nondominant hand grip strength, and RCFT delayed recall. For each pairwise association, the partial correlation coefficient was calculated after adjustment for all other variables included in the matrix. These analyses were used descriptively to examine the direction and relative magnitude of partial associations across the measures included in our analyses.

## 3 Results

Results of the regression models for each outcome are summarized in Tables 2–4, including estimates for age, sex, and education covariates. Regression analyses showed that mean trial time was a significant predictor of both Grooved Pegboard performance and nondominant hand grip strength. For Grooved Pegboard performance (Table 2), longer mean trial times were associated with poorer pegboard performance, reflected by an approximately 2-s (1.9%) increase in expected completion time for each additional second increase in mean trial time (*β* = 0.019, 95% CI = [0.007, 0.030], *p* = 0.002). In contrast, trial time variability did not significantly predict Grooved Pegboard performance (*β* = −0.015, 95% CI = [−0.056, 0.027], *p* = 0.489).

**Table 2:** Gamma generalized linear model estimates for grooved pegboard completion time.

Predictor	*β*	*SE*	95% CI	*p*
Age	0.025	0.008	[0.009, 0.041]	0.003
Sex	−0.065	0.089	[−0.245, 0.115]	0.468
Education	−0.009	0.018	[−0.045, 0.027]	0.600
Mean trial time (s)	0.019	0.006	[0.007, 0.030]	0.002
Trial time variability (s)	−0.015	0.021	[−0.056, 0.027]	0.489

SE = standard error; CI = confidence interval.

**Table 3:** Gamma generalized linear model estimates for nondominant hand grip strength.

Predictor	*β*	*SE*	95% CI	*p*
Age	−0.009	0.007	[−0.023, 0.005]	0.210
Sex	0.567	0.079	[0.407, 0.727]	<0.001
Education	−0.009	0.016	[−0.041, 0.023]	0.591
Mean trial time (s)	−0.012	0.005	[−0.022, −0.002]	0.019
Trial time variability (s)	−0.015	0.018	[−0.052, 0.022]	0.425

SE = standard error; CI = confidence interval.

**Table 4:** Gaussian generalized linear model estimates for RCFT delayed recall.

Predictor	*β*	*SE*	95% CI	*p*
Age	−0.043	0.173	[−0.382, 0.296]	0.803
Sex	2.399	1.959	[−1.440, 6.238]	0.227
Education	0.739	0.392	[−0.029, 1.507]	0.066
Mean trial time (s)	−0.143	0.124	[−0.385, 0.100]	0.256
Trial time variability (s)	−0.806	0.456	[−1.700, 0.088]	0.085

SE = standard error; CI = confidence interval.

A similar pattern was observed for grip strength (Table 3), such that longer mean trial times were associated with lower grip strength (*β* = −0.012, 95% CI = [−0.022, −0.002], *p* = 0.019), corresponding to an approximately 0.32-kg decrease in expected grip strength per one-second increase in mean trial time. In contrast, trial time variability did not significantly predict grip strength (*β* = −0.015, 95% CI = [−0.052, 0.022], *p* = 0.425).

For RCFT delayed recall, no predictors reached statistical significance based on an α = 0.05 (Table 4). However, it is important to note that the direction of estimates differed from the regression models for Grooved Pegboard and grip strength. Specifically, whereas mean trial time was positively associated with these motor outcomes, both metrics showed negative (though non-significant) estimates for RCFT delayed recall (trial time variability: *β* = −0.806, 95% CI = [−1.700, 0.088], *p* = 0.085; mean trial time: *β* = −0.143, 95% CI = [−0.385, 0.100], *p* = 0.256). Because these estimates are derived from a model with a different link function than the models for Grooved Pegboard and grip strength, their magnitudes are not directly comparable and are reported only descriptively.

As noted in 2.1 Participants, all but one participant also had WAIS-IV PRI composite scores within the normative range (mean: 100; range: 85–115). Results reported above and shown in Tables 2–4 include all participants; sensitivity analyses without this participant (PRI composite score = 84) are included in the Supplementary Tables S4–S6.

The partial correlation matrix shown in Supplementary Table S7 was consistent with the regression results. Specifically, mean trial time was positively correlated with Grooved Pegboard time (partial *r* = 0.354) and negatively correlated with nondominant hand grip strength (partial *r* = −0.213) but showed little relation to RCFT delayed recall (partial *r* = −0.080). In contrast, trial time variability showed the opposite pattern, with its largest adjusted association being with RCFT delayed recall (partial *r* = −0.279) rather than with Grooved Pegboard time (partial *r* = −0.191) or grip strength (partial *r* = −0.110). These partial correlations are computed within the same sample and adjusted for the same covariates, and their magnitudes are compared descriptively rather than tested statistically; the interpretation provided below is based on the directional pattern within each scoring method across outcomes rather than on differences in coefficient magnitude.

## 4 Discussion

This retrospective analysis used existing available data to examine whether scores derived from mean trial time and trial time variability on a functional fine motor test were differentially associated with motor and cognitive performance in older adults. The generalized linear models showed that mean trial time was significantly associated with both measures of motor function, whereas trial time variability was not. While RCFT delayed recall was not significantly associated with either mean trial time or trial time variability in the generalized linear models, partial correlation patterns differed between the two scoring methods.

Mean trial time on this functional fine motor test appears to reflect motor execution processes including manual dexterity, motor speed, and force production (Seidler et al., 2010). Prior work has shown that these aspects of motor function are affected in older adults with cognitive impairment compared to those without cognitive impairment (Wang et al., 2023; Koppelmans et al., 2024). However, cross-sectional comparisons between cognitively-impaired and -unimpaired older adults do not necessarily imply that grip dynamometry or pegboard tests should be used as a biomarker of or proxy for cognitive impairment (as has been suggested) (McGrath et al., 2019; Schubert et al., 2024). Instead, such comparisons demonstrate that individuals with cognitive impairment may, on average, be weaker or less dexterous (i.e., frailer) than those without cognitive impairment. A more critical question to address is the extent to which such motor assessments could predict the onset of future cognitive decline and eventual dementia, identifying individuals at higher risk. As such, recent work has shown that a functional fine motor test (different than the one presented here) outperformed grip strength and gait speed in predicting eventual dementia over nearly a decade (Schaefer S. Y. et al., 2025) and may be more sensitive to early cognitive and functional changes than the Mini-Mental State Examination (Schaefer S. et al., 2025).

Consistent with this, it is noted that mean trial time across six trials of the functional fine motor test used here did not predict cognitive or functional change over a shorter timespan (1 year) in older adults across the Alzheimer’s disease spectrum, whereas trial time variability did (Koppelmans et al., 2025). In the current study, trial time variability was not significantly associated with grip strength, Grooved Pegboard completion time, or RCFT delayed recall. Although this marginal relationship with the RCFT should be interpreted cautiously on its own based on the *p*-value (*p* = 0.085) in this modest sample size, the direction of the association is consistent with prior work that included a more extensive battery of neuropsychological assessments in a larger and cognitively impaired sample (Malek-Ahmadi et al., 2023). Moreover, in patients with amnestic Mild Cognitive Impairment (a prodrome of Alzheimer’s disease) and mild dementia due to Alzheimer’s disease, only trial time variability was significantly associated with hippocampal volume (a critical structure for learning and memory, as well as visuospatial processing); mean trial time was not (Schaefer et al., 2022). Furthermore, sensitivity analyses in this prior study indicated that trial time variability likely reflects learning rather than motor ability, consistent with previous literature (Müller and Sternad, 2009; Shmuelof et al., 2012). Although the present study did not include biomarkers of Alzheimer’s disease nor evaluate dementia risk, future studies should examine how different motor performance metrics relate to imaging and fluid biomarkers in clinically characterized samples.

Although demographic effects were not the primary focus of the present study, the observed associations with age, sex, and education provide useful context for interpreting the clinical measures. Overall, these patterns were consistent with prior studies showing that demographic characteristics influence scores on established motor and cognitive measures (Scheffels et al., 2023). Specifically: (1) older age was associated with poorer fine motor performance on the grooved pegboard, (2) males showed greater grip strength than females, and (3) higher educational attainment was related to better memory performance on RCFT delayed recall; all of these patterns that have all been reported in prior normative and clinical neuropsychological studies (Massy-Westropp et al., 2011; Tsatali et al., 2022; Giguère et al., 2025). These expected demographic trends suggest that the measures analyzed in the present sample are consistent with clinical literature, providing converging support for the interpretability of the primary findings.

This study has several limitations. First, because both the Grooved Pegboard Test and the functional fine motor test were scored using completion time, their association may partly reflect shared performance-speed characteristics rather than fine motor function specifically. Grooved Pegboard performance may also involve attention and executive control and should therefore not be considered as purely a measure of motor function (Bezdicek et al., 2014; Tolle et al., 2020). However, mean trial time was also significantly associated with grip strength, a non-timed measure of force production, suggesting that the observed pattern was not entirely attributable to shared timing or cognitive demands. Second, the range of RCFT delayed recall scores was likely restricted (i.e., ceiling effects) due to the fact that participants had normal scores on the WAIS-IV PRI; this likely contributed to its marginal associations with trial time variability due to a lack of dynamic range. Third, this study involved retrospective analyses of existing data collected in a systematic order that were collected as part of a different study; thus, this modest sample size was not designed *a priori* to necessarily test our current hypotheses. A sensitivity power analysis indicated that, with the present sample size (*n* = 48) and five predictors per model, the study had 80% power to detect partial associations of approximately *r* ≥ 0.38 (*f*^2^ = 0.17, *α* = 0.05). Thus, although the sample was sufficient to detect moderate effects, smaller yet potentially meaningful associations may not have been detected reliably. Larger prospective studies are therefore needed to evaluate smaller associations. The retrospective design using existing data also limited the available motor and cognitive measures, as a comprehensive neuropsychological battery was not included in the original study (NIH R03AG056822). Future prospective studies should include a broader range of motor assessments, such as finger tapping, gait and balance assessments, and other upper-limb measures, as well as tests spanning multiple cognitive domains, to determine whether the observed association patterns generalize across motor and cognitive constructs. A minor limitation also based on the use of existing data is that the observed correlations (or lack thereof) could be possibly confounded by the non-randomized order the tests were given; it is noted, however, that many existing datasets in aging (e.g., National Alzheimer’s Coordinating Center, NACC) collect their assessments in a systematic order. Lastly, we acknowledge that the RCFT is a performance-based assessment in which individuals draw their responses (first copied, and then recalled from memory), meaning that scores on this test may be impacted by motor impairment as well. While partial correlations indicated that the RCFT had weak associations with grip strength and Grooved Pegboard completion times in this study, future studies should control for motor deficits when interpreting RCFT scores, particularly in individuals with Parkinson’s disease or post-stroke hemiparesis, for example.

In conclusion, mean trial time on the functional fine motor test was significantly associated with Grooved Pegboard completion time and grip strength, whereas trial time variability was not significantly associated with either motor outcome. Neither scoring method was significantly associated with RCFT delayed recall at an α = 0.05. Consistent with broader work highlighting heterogeneity in performance-based measures among older adults (Nguyen et al., 2021; Fortinsky et al., 2026), these findings underscore the potential value of considering multiple scoring methods while interpreting each cautiously. Since the present study was not designed to evaluate clinical, diagnostic, or screening utility, larger prospective studies incorporating broader motor and cognitive assessments are needed to determine the constructs captured by trial time variability and whether either scoring method has clinical relevance.

## Data Availability

The datasets presented in this study can be found in online repositories. These data are publicly available in the Zenodo repository at https://doi.org/10.5281/zenodo.21696342.
