# Supplementary material for: Average performance and performance variability on a brief functional fine motor test reflect distinct behavioral constructs in aging
Source: Front Aging Neurosci. 2026 Sep 22;18:1957422. doi: 10.3389/fnagi.2026.1957422 (PMC13638512; doi:10.3389/fnagi.2026.1957422)
Supplement: Supplementary file 1 [file Data_Sheet_1.DOCX]

Supplementary Material

# 1 Supplementary Figures and Tables

## 1.1 Supplementary Figures

**Supplementary Figure S1.** Set-up for the functional fine motor test. Image is adapted from the “Dexterity and Reaching Motor Tasks” by MRL Laboratory that is licensed under CC BY 2.0. The experimenter instructions to participant followed this script: “*We will start with your hands on either side of the board. For this task you will use the spoon next to the board to take 2 beans out of this home cup (point to cup) and deposit them into the following pattern (point out the pattern starting with the cup on their non-dominant side), returning to the home cup before depositing the next 2 beans. Continue this pattern until you are out of beans. If you drop any beans, please continue on as we are recording for time. I will start the timer when you pick up the spoon and will stop the timer once the last 2 beans have been deposited into the last cup.  We would like you to move as fast as you can while still completing the task. Are you ready? I will start the timer as soon as you pick up the spoon.*”

**Supplementary Figure S2.** Associations of functional fine motor test scoring measures with motor and cognitive outcomes. Columns represent mean trial time and trial time variability; rows represent Grooved Pegboard completion time, nondominant hand grip strength, and Rey–Osterrieth Complex Figure Test (RCFT) delayed recall score. Blue points represent observed values. Red lines and shaded areas represent model-estimated associations and their 95% confidence intervals, respectively. The displayed β coefficients and p values were obtained from generalized linear models adjusted for age, sex, education, and the other functional fine motor test scoring measure. Grooved Pegboard completion time and grip strength were modeled using Gamma distributions with log links, whereas RCFT delayed recall was modeled using a Gaussian distribution with an identity link. No non-linear terms were included; fitted curves for Grooved Pegboard completion time and grip are back-transformations of the linear predictions on a logarithmic scale to the original outcome scale for visualization purposes.

## Supplementary Tables

**Supplementary Table S1.** Trial 1 sensitivity analysis: Gamma generalized linear model estimates for grooved pegboard completion time.

| **Predictor** | **β** | ***SE*** | **95% CI** | ***p*** |
| --- | --- | --- | --- | --- |
| **Age** | 0.026 | 0.008 | [0.009, 0.042] | 0.004 |
| **Sex** | -0.070 | 0.092 | [-0.251, 0.111] | 0.451 |
| **Education** | -0.014 | 0.018 | [-0.050, 0.022] | 0.444 |
| **Trial 1 time (s)** | 0.015 | 0.005 | [0.004, 0.026] | 0.008 |
| **ISD in trial time (s)** | -0.026 | 0.024 | [-0.072, 0.021] | 0.291 |

*Note.* Trial 1 time replaced mean trial time in the original model. ISD = intrasubject standard deviation; SE = standard error; CI = confidence interval.

**Supplementary Table S2.** Trial 1 sensitivity analysis: Gamma generalized linear model estimates for nondominant-hand grip strength.

| **Predictor** | **β** | ***SE*** | **95% CI** | ***p*** |
| --- | --- | --- | --- | --- |
| **Age** | -0.009 | 0.007 | [-0.023, 0.005] | 0.214 |
| **Sex** | 0.566 | 0.080 | [0.410, 0.723] | <0.001 |
| **Education** | -0.006 | 0.016 | [-0.037, 0.025] | 0.709 |
| **Trial 1 time (s)** | -0.011 | 0.005 | [-0.020, -0.002] | 0.027 |
| **ISD in trial time (s)** | -0.004 | 0.021 | [-0.045, 0.037] | 0.847 |

*Note.* Trial 1 time replaced mean trial time in the original model. ISD = intrasubject standard deviation; SE = standard error; CI = confidence interval.

**Supplementary Table S3.** Trial 1 sensitivity analysis: Gaussian generalized linear model estimates for RCFT delayed recall.

| **Predictor** | **β** | ***SE*** | **95% CI** | ***p*** |
| --- | --- | --- | --- | --- |
| **Age** | -0.095 | 0.179 | [-0.446, 0.257] | 0.600 |
| **Sex** | 2.391 | 1.986 | [-1.500, 6.283] | 0.235 |
| **Education** | 0.782 | 0.395 | [0.008, 1.557] | 0.054 |
| **Trial 1 time (s)** | -0.046 | 0.116 | [-0.274, 0.181] | 0.691 |
| **ISD in trial time (s)** | -0.889 | 0.515 | [-1.898, 0.119] | 0.091 |

*Note.* Trial 1 time replaced mean trial time in the original model. ISD = intrasubject standard deviation; SE = standard error; CI = confidence interval; RCFT = Rey–Osterrieth Complex Figure Test.

**Supplementary Table S4.** PRI-exclusion sensitivity analysis: Gamma generalized linear model estimates for grooved pegboard completion time.

| **Predictor** | **β** | ***SE*** | **95% CI** | ***p*** |
| --- | --- | --- | --- | --- |
| **Age** | 0.025 | 0.008 | [0.009, 0.041] | 0.003 |
| **Sex** | -0.066 | 0.091 | [-0.244, 0.112] | 0.474 |
| **Education** | -0.009 | 0.018 | [-0.045, 0.026] | 0.604 |
| **Mean trial time (s)** | 0.019 | 0.006 | [0.007, 0.030] | 0.002 |
| **ISD in trial time (s)** | -0.015 | 0.022 | [-0.057, 0.028] | 0.502 |

*Note.* The participant with PRI composite score = 84 was excluded (n = 47). ISD = intrasubject standard deviation; SE = standard error; CI = confidence interval; PRI = Perceptual Reasoning Index.

**Supplementary Table S5.** PRI-exclusion sensitivity analysis: Gamma generalized linear model estimates for nondominant-hand grip strength.

| **Predictor** | **β** | ***SE*** | **95% CI** | ***p*** |
| --- | --- | --- | --- | --- |
| **Age** | -0.008 | 0.007 | [-0.022, 0.005] | 0.231 |
| **Sex** | 0.557 | 0.079 | [0.402, 0.711] | <0.001 |
| **Education** | -0.011 | 0.016 | [-0.042, 0.020] | 0.504 |
| **Mean trial time (s)** | -0.012 | 0.005 | [-0.022, -0.003] | 0.016 |
| **ISD in trial time (s)** | -0.020 | 0.019 | [-0.057, 0.017] | 0.293 |

*Note.* The participant with PRI composite score = 84 was excluded (n = 47). ISD = intrasubject standard deviation; SE = standard error; CI = confidence interval; PRI = Perceptual Reasoning Index.

**Supplementary Table S6.** PRI-exclusion sensitivity analysis: Gaussian generalized linear model estimates for RCFT delayed recall.

| **Predictor** | **β** | ***SE*** | **95% CI** | ***p*** |
| --- | --- | --- | --- | --- |
| **Age** | -0.045 | 0.175 | [-0.388, 0.298] | 0.799 |
| **Sex** | 2.439 | 1.994 | [-1.469, 6.346] | 0.228 |
| **Education** | 0.747 | 0.399 | [-0.035, 1.529] | 0.068 |
| **Mean trial time (s)** | -0.143 | 0.125 | [-0.388, 0.103] | 0.261 |
| **ISD in trial time (s)** | -0.786 | 0.474 | [-1.715, 0.143] | 0.105 |

*Note.* The participant with PRI composite score = 84 was excluded (n = 47). ISD = intrasubject standard deviation; *SE* = standard error; CI = confidence interval; PRI = Perceptual Reasoning Index; RCFT = Rey–Osterrieth Complex Figure Test.

**Supplementary Table S7.** Partial correlation matrix.

| **Variable** | **Age (yrs)** | **Sex^a^** | **Education (yrs)** | **Mean trial time (s)** | **Trial time variability (s)** | **Grooved pegboard (s)** | **Grip strength (kg)** | **RCFT delayed recall** |
| --- | --- | --- | --- | --- | --- | --- | --- | --- |
| **Age (yrs)** | — | 0.197 | -0.150 | 0.149 | 0.145 | 0.381 | -0.030 | 0.030 |
| **Sex** | 0.197 | — | 0.149 | 0.208 | 0.053 | 0.149 | 0.754 | 0.097 |
| **Education (yrs)** | -0.150 | 0.149 | — | -0.073 | 0.124 | -0.003 | -0.078 | 0.279 |
| **Mean trial time (s)** | 0.149 | 0.208 | -0.073 | — | 0.316 | 0.354 | -0.213 | -0.080 |
| **Trial time variability (s)** | 0.145 | 0.053 | 0.124 | 0.316 | — | -0.191 | -0.110 | -0.279 |
| **Grooved pegboard (s)** | 0.381 | 0.149 | -0.003 | 0.354 | -0.191 | — | -0.278 | -0.148 |
| **Grip strength (kg)** | -0.030 | 0.754 | -0.078 | -0.213 | -0.110 | -0.278 | — | 0.017 |
| **RCFT delayed recall** | 0.030 | 0.097 | 0.279 | -0.080 | -0.279 | -0.148 | 0.017 | — |

*Note.* Values are partial correlation coefficients, *r*. ^a^Sex was coded as male = 1 and female = 0. Grooved pegboard = nondominant grooved pegboard completion time; Grip strength = nondominant hand grip strength; RCFT = Rey–Osterrieth Complex Figure Test.
